# Counting the Minutes

**DOI:** 10.7554/eLife.53348

**Published:** 2020-01-07

**Authors:** Stephano Mello, Dirk Bohmann

**Affiliations:** Department of Biomedical GeneticsUniversity of Rochester Medical CenterRochesterUnited States

**Keywords:** cell competition, ribosomal protein, xrp1, growth control, genetic mutations, *D. melanogaster*

## Abstract

A newly discovered mechanism that causes the 'Minute' phenotype in fruit flies can explain how organisms are able to eliminate the mutant cells that arise occasionally during development.

**Related research article** Blanco J, Cooper JC, Baker NE. 2019. Roles of C/EBP class bZip proteins in the growth and cell competition of rp ('Minute') mutants in *Drosophila*. *eLife* **8**:e50535. doi: 10.7554/eLife.50535

Almost exactly 100 years ago Calvin Bridges, a member of the famous ‘Flyroom’ in Thomas Hunt Morgan's lab at Columbia University, made an observation that is still having an impact to this day. Bridges discovered a genetic mutation that caused the bristles on the back of fruit flies to be slightly thinner and shorter than those of wild-type flies (Bridges and Morgan, 1923). Bridges also noticed that these mutants, which he named ‘Minute’ after their small bristle phenotype, took a day and a half longer than normal to develop. Once he knew what to look for, Bridges was astounded to find many more mutants with the same characteristics. Within a year, another 25 mutations that cause the Minute phenotype had been found randomly scattered across the fly genome, with the final tally reaching 50 different mutation sites.

It is now known that the reason there are so many Minute mutants is because they are caused by disruptions in one of approximately 65 Rp genes that code for ribosomal proteins (Marygold et al., 2007). These proteins are essential building blocks of ribosomes – the factories that make proteins in living cells. The Minute phenotype that Bridges observed was caused by a heterozygous mutation (mutation in one of two parental alleles) in a single Rp gene: such mutations are compatible with a healthy life, whereas complete loss of the gene (i.e. a mutation in both alleles) disrupts ribosome function and results in death.

One of the intriguing biological discoveries arising from studies of Minute mutants is the phenomenon of ‘cell competition’ (Baker, 2017; Gogna et al., 2015; Morata and Ripoll, 1975; Simpson and Morata, 1981). Embryos with a heterozygous Minute mutation (Rp^+/-^) develop into normal-sized adult fruit flies with no apparent defects (except for the small bristles). However, in genetically mosaic animals, when an Rp^+/-^ cell comes into contact with wildtype cells that have two intact copies of the affected Rp gene (Rp^+/+^), the mutant cell dies. This juxtaposition of Rp^+/-^ and wildtype cells may result from random cell mutations occurring during development. The mechanism of cell competition allows developing organisms to eliminate cells with an incomplete set of Rp genes, as long as other genetically intact (+/+) cells are available (reviewed in Baker, 2017).

How Rp^+/-^ cells are eliminated during development has long remained a mystery. The most obvious explanation was that having fewer ribosomes or reduced ribosome function results in fewer proteins being made, which leads to the Rp^+/-^ cells being outcompeted by the wild-type cells surrounding them. Now, in eLife, Jorge Blanco (Albert Einstein College of Medicine), Jacob Cooper (University of Utah) and Nicholas Baker (Albert Einstein College) report new insights into cell competition in fruit flies (Blanco et al., 2019).

Blanco et al. showed that the decreased growth and reduced protein synthesis in Rp^+/-^ cells was dependent on a transcription factor called Xrp1 (Kale et al., 2018; Lee et al., 2018). Normally, a group of Rp^+/-^ cells will make fewer proteins, grow slower and ultimately undergo apoptosis. However, if this group of cells lack the Xrp1 transcription factor, none of these things happen (Figure 1A). Instead, Blanco et al. conclude that all the distinctive phenotypes of Minute mutant cells, in otherwise wildtype organisms, are the result of a regulatory mechanism that senses differences in the status of Rp genes between adjacent cells. This then triggers gene expression programs that ultimately cause the demise of Rp^+/-^ cells (Figure 1B).

**Figure 1.**
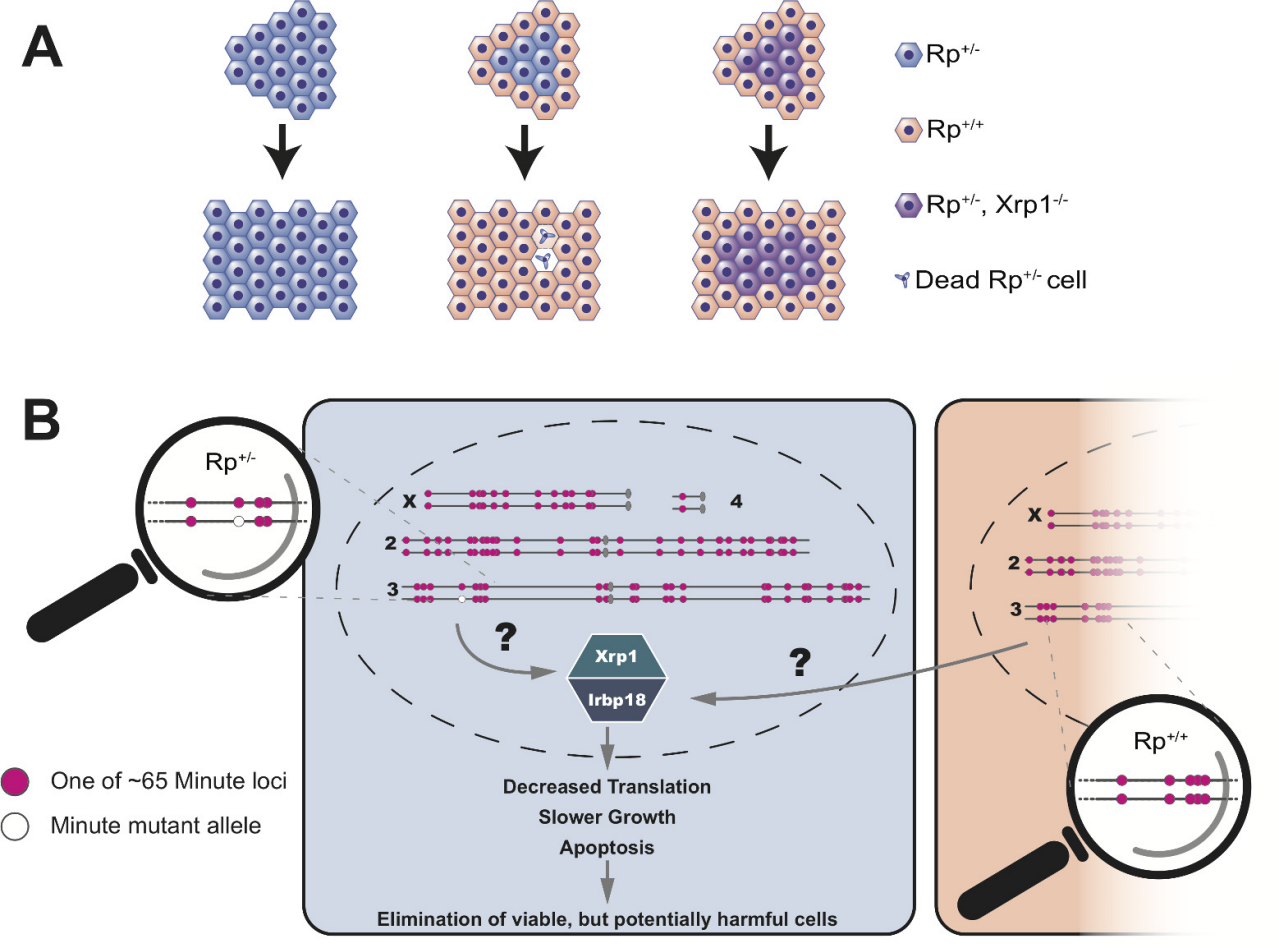
Cell competition is driven by differences in the number of Rp genes. (**A**) A tissue that consists entirely of cells in which one of the Rp genes is present in only one copy (Rp^+/-^, shown in light blue) grows to a normal size. However, Rp^+/-^ cells are outcompeted and eliminated by apoptosis when they are adjacent to wildtype cells (Rp^+/+^, orange). The loss of Rp^+/-^ cells depends on a transcription factor called Xrp1: if cells lack one Rp allele and are also mutant for Xrp1 (Rp^+/-^, Xrp1^-/-^, shown in purple) they will not be eliminated. (**B**) The Rp genes are spread out over all four chromosomes of the fruit fly. About 65 of these genes (red dots) cause the Minute phenotype when only one copy is present (one red dot across from one white dot). When a heterozygous cell, in which any of these 65 genes is present in only one copy (Rp^+/-^), is located adjacent to a cell with a complete set of Rp genes (Rp^+/+^), this triggers the expression of Xrp1 and its binding partner Irbp18. The increased activity of this Xrp1/Irbp18 dimer is required for the effects of cell competition: decreased protein synthesis, slower growth and ultimately apoptosis in the Rp^+/-^ cells.

These findings have a number of important implications. First, heterozygous mutations in human ribosome genes can cause serious diseases, such as Diamond-Blackfan anemia (which leads to a lack of red blood cells and developmental defects). Second, cell competition occurs in other organisms, including mammals: for example, cancer cells that are deficient for the tumor suppressor gene p53 can be outcompeted by cells in which p53 is still intact (Baker et al., 2019). Notably, the Xrp1 transcription factor that Blanco et al. identified in flies is also a p53 target gene, suggesting that tumor growth may rely on a similar mechanism of cell competition. Third, there are more than 50 Rp genes that represent potential Minute mutation sites scattered throughout the fruit fly and human genomes, and cells missing any one of these alleles can be eliminated by the Xrp1-dependent mechanism. This suggests that sensing of Rp heterozygosity could be used by genome surveillance mechanisms to detect more complex mutations (e.g. aneuploidies or large deletions) that involve Rp genes. Therefore, Rp mutations could act as an indicator for detrimental chromosome defects that are known to occur in cancer progression and during normal development.

Although it is clear how important a Xrp1-related mechanism could be in mammals, finding it turned out to be unexpectedly difficult. This is because the Xrp1 gene appears to undergo rapid evolutionary change and initial attempts to identify orthologs in insect species other than fruit flies – let alone in humans – failed. However, Blanco et al. discovered that Xrp1 has an essential binding partner called lrbp18 that has recognizable relatives in mammalian cells. By identifying human proteins that can form complexes with lrbp18, they were able to find potential genes that correspond to Xrp1 in humans. This discovery opens up exciting perspectives for studying the role of Xrp1-like genes, and their possible interplay with p53 in cancer and genome surveillance.

Pioneering work that was published over 100 years ago by Bridges, and other scientists in the fruit fly community still provides the basis for potentially groundbreaking discoveries in biology and medicine. This story illustrates one of the inspiring aspects of science – that inauspicious observations, like a slightly shorter bristle on the back of a fly, can lead to profound impacts on human medicine.
